# Rb_2_Sb_4_O_11_

**DOI:** 10.1107/S1600536809016109

**Published:** 2009-05-07

**Authors:** Govindaraasan Kalpana, Mark T. Weller

**Affiliations:** a School of Chemistry, University of Southampton, Highfield Campus, Southamtpon SO17 1BJ, England

## Abstract

The title compound, dirubidium tetra­anti­monate(V), Rb_2_Sb_4_O_11_, has been synthesized by flux reaction. It is isotypic with known *A*_2_Sb_4_O_11_ (*A* = K, Cs) structures and consists of an (Sb_4_O_11_)^2−^ skeleton and two Rb atoms as charge-compensating cations. Distorted SbO_6_ octa­hedra share edges and corners, resulting in a layered assembly. Alternate stacking of the layers along the *c* axis leads to the formation of tunnels. The Rb^+^ ions, surrounded by nine and ten O atoms, respectively, are located in these tunnels. Some atoms in the structure are on special positions of *m* symmetry (two Sb atoms, both Rb atoms and four O atoms) and 2 symmetry (one O atom).

## Related literature

Isotypic structures have been reported by Hong (1974 ▶) [K_2_Sb_4_O_11_] and by Hirschle *et al.* (2001 ▶) [Cs_2_Sb_4_O_11_]. For Rb—O distances in the crystal structure of Rb_3_Ti_2_(TiO)(PO_4_)_3_P_2_O_7_, see: Duhlev (1994 ▶).

## Experimental

### 

#### Crystal data

Rb_2_Sb_4_O_11_
*M* *_r_* = 833.94
Monoclinic, 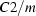

*a* = 19.5045 (11) Å
*b* = 7.5681 (4) Å
*c* = 7.2115 (4) Å
β = 95.203 (3)°
*V* = 1060.12 (10) Å^3^
*Z* = 4
Mo *K*α radiation
μ = 19.26 mm^−1^
*T* = 120 K
0.12 × 0.12 × 0.11 mm

#### Data collection

Nonius KappaCCD diffractometer
Absorption correction: multi-scan (*SADABS*; Sheldrick, 2007 ▶) *T*_min_ = 0.110, *T*_max_ = 0.120
7605 measured reflections
1312 independent reflections
1234 reflections with *I* > 2σ(*I*)
*R*_int_ = 0.038

#### Refinement

*R*[*F* ^2^ > 2σ(*F* ^2^)] = 0.028
*wR*(*F* ^2^) = 0.072
*S* = 1.41
1312 reflections
91 parameters
18 restraints
Δρ_max_ = 1.89 e Å^−3^
Δρ_min_ = −1.91 e Å^−3^

### 

Data collection: *COLLECT* (Nonius, 1998 ▶); cell refinement: *DENZO* (Otwinowski & Minor, 1997 ▶) and *COLLECT*; data reduction: *DENZO* nd *COLLECT*; program(s) used to solve structure: *SHELXS97* (Sheldrick, 2008 ▶); program(s) used to refine structure: *SHELXL97* (Sheldrick, 2008 ▶); molecular graphics: *DIAMOND* (Brandenburg, 2006 ▶); software used to prepare material for publication: *WinGX* (Farrugia, 1999 ▶).

## Supplementary Material

Crystal structure: contains datablocks global, I. DOI: 10.1107/S1600536809016109/wm2230sup1.cif

Structure factors: contains datablocks I. DOI: 10.1107/S1600536809016109/wm2230Isup2.hkl

Additional supplementary materials:  crystallographic information; 3D view; checkCIF report

## Figures and Tables

**Table 1:** Selected bond lengths (Å)

Sb1—O4	1.940 (4)
Sb1—O3^i^	1.956 (5)
Sb1—O1^ii^	1.986 (3)
Sb1—O2	2.089 (5)
Sb2—O3^iii^	1.903 (5)
Sb2—O6^iv^	1.970 (3)
Sb2—O2^v^	1.977 (5)
Sb2—O5^iii^	1.993 (5)
Sb2—O2^iii^	2.129 (5)
Sb3—O8	1.9281 (15)
Sb3—O4	1.959 (4)
Sb3—O7^vi^	1.978 (4)
Sb3—O7	1.979 (4)
Sb3—O6	2.005 (4)
Sb3—O5	2.0261 (14)

Symmetry codes: (i) 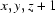; (ii) 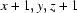; (iii) 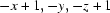; (iv) 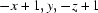; (v) 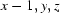; (vi) 
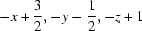
.

**Fig. 1.**
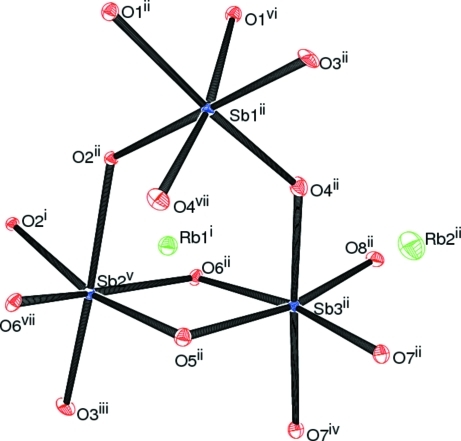
ORTEP plot of the asymmetric unit of compound (1) Thermal ellipsoids are given at the 50% probability level. [Symmetry codes: (i) x, -y, z; (ii) -x + 2, -y, -z + 2; (iii) -x + 2, -y - 1, -z + 2; (iv) -x + 2, y - 1, -z + 1; (v) -x + 2, -y - 1, -z + 1; (vi) x, y - 1, z; (vii) -x + 3/2, -y - 1/2, -z + 1].

**Fig. 2.**
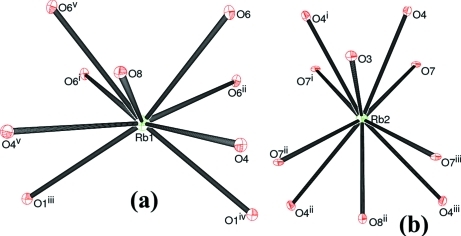
ORTEP diagrams of the coordination environment of (a) Rb1 and (b) Rb2 atoms of compound (1). Displacement ellipsoids are drawn at the 50% probability level. [Symmetry codes (i) x, -y, z; (ii) -x + 2, -y, -z + 2; iii) -x + 2, -y - 1, -z + 2; (iv) -x + 2, y - 1, -z + 1; (v) -x + 2, -y - 1, -z + 1].

**Fig. 3.**
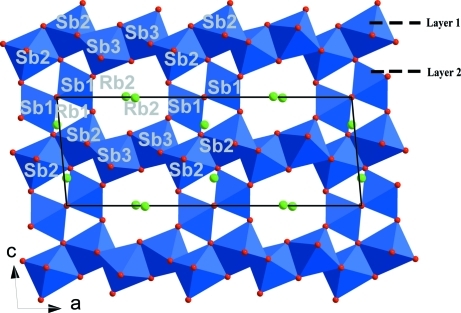
Polyhedral representation of compound (1) along the ac plane: blue octahedra, red spheres, green spheres represent SbO6, O and Rb atoms respectively

## supplementary crystallographic information

### Comment

Single crystals of the title compound (1), were formed inadvertently during one of our flux syntheses aimed at producing new oxides in the quaternary Rb/Sb/B/O system. There are two *A*_2_Sb_4_O_11_ (*A* = K, Cs) compounds known and their structures were determined by single-crystal X-ray diffraction (Hong, 1974 and Hirschle *et al.*, 2001). *A*_2_Sb_4_O_11_(*A* = K, Cs) crystallize in the centrosymmetric space group *C*2/*m* and have two-dimensional structures with Sb in octahedral coordination. In K_2_Sb_4_O_11_, the K^+^ ions are mobile in the tunnels. The K^+^ ions have been ion-exchanged with Na^+^, Ag^+^, Rb^+^ and TI^+^ in molten salts, but neither their unit-cell parameters nor their crystal structures are available (Hong, 1974). In Cs_2_Sb_4_O_11_, Cs^+^ ions are not mobile (Hirschle *et al.*, 2001). Here, we report the crystal structure of Rb_2_Sb_4_O_11_, confirming that it is isotypic with *A*_2_Sb_4_O_11_ (*A* = K, Cs).

The crystal structure of compound (1) contains two Rb (Rb1 and Rb2), three Sb (Sb1, Sb2 and Sb3) and eight O (O1—O8) atoms (Fig.1). The atoms Sb1, O4, O6 and O7 are on general positions, all other atoms are on special positions, viz. mirror planes and twofold-rotation axes. Each antimony atom is coordinated to six oxygen atoms to form distorted octahedra with Sb—O distances ranging from 1.903 (4) to 2.129 (5) Å, comparable to those in the isotypic antimonates(V) (Hong, 1974; Hirschle *et al.*, 2001). Rb1 is nine fold coordinated and Rb2 is ten fold coordinated to oxygen atoms (Fig. 2 (*a*) and 2(*b*)) with Rb—O distances ranging from 2.933 (4) - 3.473 (4) Å, comparable to Rb_3_Ti_2_(TiO)(PO_4_)_3_P_2_O_7_ (Duhlev, 1994). The coordination number of Rb^+^ ion differs from the the 11 coordinated A^+^ ions reported for the isotypic *A*_2_Sb_4_O_11_ (*A* = K, Cs) compounds, where the non bonding distances of K—O; 3.78 (3) - 3.832 (4) Å, Cs—O; 3.721 (8) - 3.940 (9) Å were considered as bonds.

In the asymmetric unit of the title compound (1) all oxygen atoms are shared between two SbO_6_ octahedra, except the oxygen atoms O(2) and O(4), that are common to all three Sb(1)O_6_, Sb(2)O_6_ and Sb(3)O_6_ octahedra (Fig. 1). In compound (1), there are two different layers (1 and 2) formed by edge-sharing of Sb(1)—Sb(1), Sb(2)—Sb(2) and Sb(3)—Sb(3) octahedra to form three types of Sb_2_O_10_ dimers (Fig. 3). Layer 1 is formed by edge-sharing of the Sb_2_(2)O_10_ and Sb_2_(3)O_10_ dimers and layer 2 is formed by Sb_2_(1)O_10_ dimers sharing corners with the layer 1. Alternate stacking of these two layers along the *c* axis leads to formation of tunnels and Rb^+^ ions are located in these tunnels. The single-crystal data was measured at 123 K and the displacement parameters observed for Rb^+^ are roughly isotropic, indicating that they are not mobile in the channels.

### Experimental

A mixture of Rb_2_CO_3_ (Aldrich, 0.6224 g; 2.70 mmol), Sb_2_O_3_ (Aldrich, 0.3143 g; 1.08 mmol) and H_3_BO_3_ (Aldrich,0.5000 g; 8.09 mmol) was ground in a mortar and pestle. The ground mixture was then added into a platinum crucible. The furnace temperature was slowly raised from room temperature and heated at 773 K for 12 hrs, 923 K for a further 12 hrs, and then kept at 1273 K for 24 hrs. The furnace was cooled to room temperature over a period of 48 hrs. The melt was washed with hot water to remove the excess boric acid, filtered and dried in an oven at 353 K. Colourless crystals of compound (1) were obtained from the melt.

### Refinement

All atoms were refined anisotropically. It was necessary to apply *SHELX* ISOR restraints to O2, O5 and O1; a value of 0.001 Å^2^ was used. The highest peak and the deepest hole of the final Fourier map are located at 1.85 Å from Rb1 and 0.85 Å from the Sb2 atom, respectively.

### Crystal data

**Table:**

Rb_2_Sb_4_O_11_	*F*(000) = 1464
*M**_r_* = 833.94	*D*_x_ = 5.225 Mg m^−^^3^
Monoclinic, *C*2/*m*	Mo *K*α radiation, λ = 0.71073 Å
Hall symbol: -C 2y	Cell parameters from 1307 reflections
*a* = 19.5045 (11) Å	θ = 2.9–27.5°
*b* = 7.5681 (4) Å	µ = 19.26 mm^−^^1^
*c* = 7.2115 (4) Å	*T* = 120 K
β = 95.203 (3)°	Block, colourless
*V* = 1060.12 (10) Å^3^	0.12 × 0.12 × 0.11 mm
*Z* = 4	

### Data collection

**Table:**

Nonius KappaCCD diffractometer	1312 independent reflections
Radiation source: Nonius FR591 Rotating Anode	1234 reflections with *I* > 2σ(*I*)
graphite	*R*_int_ = 0.038
Detector resolution: 9.091 pixels mm^-1^	θ_max_ = 27.6°, θ_min_ = 3.7°
φ and ω scans	*h* = −25→25
Absorption correction: multi-scan (*SADABS*; Sheldrick, 2007)	*k* = −9→9
*T*_min_ = 0.110, *T*_max_ = 0.120	*l* = −9→8
7605 measured reflections	

### Refinement

**Table:**

Refinement on *F*^2^	Primary atom site location: structure-invariant direct methods
Least-squares matrix: full	Secondary atom site location: difference Fourier map
*R*[*F*^2^ > 2σ(*F*^2^)] = 0.028	*w* = 1/[σ^2^(*F*_o_^2^) + (0.029*P*)^2^ + 1.4332*P*] where *P* = (*F*_o_^2^ + 2*F*_c_^2^)/3
*wR*(*F*^2^) = 0.072	(Δ/σ)_max_ = 0.001
*S* = 1.41	Δρ_max_ = 1.89 e Å^−^^3^
1312 reflections	Δρ_min_ = −1.91 e Å^−^^3^
91 parameters	Extinction correction: *SHELXL97* (Sheldrick, 2008), Fc^*^=kFc[1+0.001xFc^2^λ^3^/sin(2θ)]^-1/4^
18 restraints	Extinction coefficient: 0.00092 (9)

### Special details

**Table:**

Experimental. *SADABS* was used to perform the Absorption correction
Geometry. All e.s.d.'s (except the e.s.d. in the dihedral angle between two l.s. planes) are estimated using the full covariance matrix. The cell e.s.d.'s are taken into account individually in the estimation of e.s.d.'s in distances, angles and torsion angles; correlations between e.s.d.'s in cell parameters are only used when they are defined by crystal symmetry. An approximate (isotropic) treatment of cell e.s.d.'s is used for estimating e.s.d.'s involving l.s. planes.
Refinement. Refinement of *F*^2^ against ALL reflections. The weighted *R*-factor *wR* and goodness of fit *S* are based on *F*^2^, conventional *R*-factors *R* are based on *F*, with *F* set to zero for negative *F*^2^. The threshold expression of *F*^2^ > σ(*F*^2^) is used only for calculating *R*-factors(gt) *etc*. and is not relevant to the choice of reflections for refinement. *R*-factors based on *F*^2^ are statistically about twice as large as those based on *F*, and *R*- factors based on ALL data will be even larger.

### Fractional atomic coordinates and isotropic or equivalent isotropic displacement parameters (Å^2^)

**Table:**

	*x*	*y*	*z*	*U*_iso_*/*U*_eq_	
Sb1	0.92822 (2)	0.0000	0.90178 (6)	0.00457 (16)	
Sb2	0.07568 (2)	0.0000	0.61748 (6)	0.00476 (16)	
Sb3	0.825607 (17)	−0.25714 (4)	0.56390 (5)	0.00453 (15)	
Rb1	0.99190 (4)	−0.5000	0.74518 (10)	0.0115 (2)	
Rb2	0.73468 (4)	0.0000	0.00596 (10)	0.0128 (2)	
O1	0.0000	0.1704 (7)	0.0000	0.0060 (10)	
O2	0.9765 (3)	0.0000	0.6548 (7)	0.0045 (10)	
O3	0.8831 (3)	0.0000	0.1331 (7)	0.0080 (11)	
O4	0.8715 (2)	−0.1971 (5)	0.8087 (5)	0.0081 (8)	
O5	0.8339 (3)	0.0000	0.4910 (7)	0.0072 (10)	
O6	0.9112 (2)	−0.2537 (4)	0.4296 (5)	0.0061 (8)	
O7	0.7364 (2)	−0.2127 (5)	0.6665 (5)	0.0069 (8)	
O8	0.8392 (3)	−0.5000	0.6392 (7)	0.0070 (11)	

### Atomic displacement parameters (Å^2^)

**Table:**

	*U*^11^	*U*^22^	*U*^33^	*U*^12^	*U*^13^	*U*^23^
Sb1	0.0050 (3)	0.0049 (3)	0.0039 (3)	0.000	0.00008 (18)	0.000
Sb2	0.0046 (3)	0.0052 (3)	0.0045 (3)	0.000	0.00049 (18)	0.000
Sb3	0.0045 (2)	0.0042 (2)	0.0049 (2)	−0.00016 (12)	0.00030 (15)	−0.00009 (11)
Rb1	0.0108 (4)	0.0125 (4)	0.0111 (4)	0.000	0.0012 (3)	0.000
Rb2	0.0155 (4)	0.0137 (4)	0.0096 (4)	0.000	0.0027 (3)	0.000
O1	0.0054 (13)	0.0058 (13)	0.0067 (13)	0.000	0.0007 (9)	0.000
O2	0.0041 (13)	0.0048 (13)	0.0047 (13)	0.000	0.0004 (9)	0.000
O3	0.012 (3)	0.010 (3)	0.002 (2)	0.000	0.001 (2)	0.000
O4	0.011 (2)	0.0064 (18)	0.0073 (19)	−0.0017 (16)	0.0009 (15)	0.0001 (14)
O5	0.0067 (13)	0.0076 (13)	0.0073 (13)	0.000	0.0009 (9)	0.000
O6	0.0042 (19)	0.005 (2)	0.009 (2)	0.0001 (13)	0.0008 (15)	0.0010 (13)
O7	0.006 (2)	0.0095 (18)	0.0050 (18)	0.0001 (16)	0.0024 (14)	−0.0001 (14)
O8	0.009 (3)	0.006 (2)	0.006 (2)	0.000	−0.001 (2)	0.000

### Geometric parameters (Å, °)

**Table:**

Sb1—O4	1.940 (4)	Rb1—O8	3.007 (5)
Sb1—O4^i^	1.940 (4)	Rb1—O6^x^	3.011 (4)
Sb1—O3^ii^	1.956 (5)	Rb1—O6^xi^	3.011 (4)
Sb1—O1^iii^	1.986 (3)	Rb1—O1^iv^	3.094 (4)
Sb1—O1^iv^	1.986 (3)	Rb1—O1^xii^	3.094 (4)
Sb1—O2	2.089 (5)	Rb1—O6^xiii^	3.238 (4)
Sb1—Sb1^v^	3.0211 (10)	Rb1—O6	3.238 (4)
Sb2—O3^iv^	1.903 (5)	Rb1—O4^xiii^	3.343 (4)
Sb2—O6^vi^	1.970 (3)	Rb1—O4	3.343 (4)
Sb2—O6^iv^	1.970 (3)	Rb1—Rb1^xi^	3.5787 (14)
Sb2—O2^vii^	1.977 (5)	Rb1—Rb1^xiv^	3.6602 (14)
Sb2—O5^iv^	1.993 (5)	Rb2—O7^xv^	2.933 (4)
Sb2—O2^iv^	2.129 (5)	Rb2—O7^xvi^	2.933 (4)
Sb2—Sb3^iv^	3.1084 (5)	Rb2—O3	2.956 (5)
Sb2—Sb3^vi^	3.1084 (5)	Rb2—O8^ix^	3.048 (5)
Sb2—Sb2^viii^	3.2679 (10)	Rb2—O7^xvii^	3.222 (4)
Sb3—O8	1.9281 (15)	Rb2—O7^ix^	3.222 (4)
Sb3—O4	1.959 (4)	Rb2—O4^ix^	3.440 (4)
Sb3—O7^ix^	1.978 (4)	Rb2—O4^xvii^	3.440 (4)
Sb3—O7	1.979 (4)	Rb2—O4^xvi^	3.473 (4)
Sb3—O6	2.005 (4)	Rb2—O4^xv^	3.473 (4)
Sb3—O5	2.0261 (14)	Rb2—Rb2^xviii^	3.8331 (3)
Sb3—Sb3^ix^	3.0111 (7)	Rb2—Rb2^xix^	3.8331 (3)
Sb3—Sb2^iv^	3.1084 (5)		
			
O4—Sb1—O4^i^	100.5 (2)	O6^xiii^—Rb1—O6	70.31 (13)
O4—Sb1—O3^ii^	90.47 (15)	O8—Rb1—O4^xiii^	48.93 (8)
O4^i^—Sb1—O3^ii^	90.47 (15)	O6^x^—Rb1—O4^xiii^	163.20 (9)
O4—Sb1—O1^iii^	169.68 (15)	O6^xi^—Rb1—O4^xiii^	96.20 (9)
O4^i^—Sb1—O1^iii^	89.17 (16)	O1^iv^—Rb1—O4^xiii^	118.05 (7)
O3^ii^—Sb1—O1^iii^	93.05 (12)	O1^xii^—Rb1—O4^xiii^	50.52 (7)
O4—Sb1—O1^iv^	89.17 (16)	O6^xiii^—Rb1—O4^xiii^	53.05 (9)
O4^i^—Sb1—O1^iv^	169.68 (16)	O6—Rb1—O4^xiii^	100.87 (10)
O3^ii^—Sb1—O1^iv^	93.05 (12)	O8—Rb1—O4	48.93 (8)
O1^iii^—Sb1—O1^iv^	81.0 (2)	O6^x^—Rb1—O4	96.20 (9)
O4—Sb1—O2	89.55 (13)	O6^xi^—Rb1—O4	163.20 (9)
O4^i^—Sb1—O2	89.55 (14)	O1^iv^—Rb1—O4	50.52 (7)
O3^ii^—Sb1—O2	180.0 (2)	O1^xii^—Rb1—O4	118.05 (7)
O1^iii^—Sb1—O2	86.92 (11)	O6^xiii^—Rb1—O4	100.87 (10)
O1^iv^—Sb1—O2	86.92 (11)	O6—Rb1—O4	53.05 (9)
O4—Sb1—Sb1^v^	129.58 (11)	O4^xiii^—Rb1—O4	86.60 (13)
O4^i^—Sb1—Sb1^v^	129.58 (11)	O7^xv^—Rb2—O7^xvi^	66.58 (15)
O3^ii^—Sb1—Sb1^v^	94.01 (16)	O7^xv^—Rb2—O3	100.00 (11)
O1^iii^—Sb1—Sb1^v^	40.48 (11)	O7^xvi^—Rb2—O3	100.00 (11)
O1^iv^—Sb1—Sb1^v^	40.48 (11)	O7^xv^—Rb2—O8^ix^	137.98 (10)
O2—Sb1—Sb1^v^	85.95 (14)	O7^xvi^—Rb2—O8^ix^	137.98 (10)
O3^iv^—Sb2—O6^vi^	96.46 (12)	O3—Rb2—O8^ix^	105.29 (14)
O3^iv^—Sb2—O6^iv^	96.46 (12)	O7^xv^—Rb2—O7^xvii^	165.42 (8)
O6^vi^—Sb2—O6^iv^	154.0 (2)	O7^xvi^—Rb2—O7^xvii^	103.13 (8)
O3^iv^—Sb2—O2^vii^	101.9 (2)	O3—Rb2—O7^xvii^	70.82 (10)
O6^vi^—Sb2—O2^vii^	99.63 (12)	O8^ix^—Rb2—O7^xvii^	56.58 (9)
O6^iv^—Sb2—O2^vii^	99.63 (12)	O7^xv^—Rb2—O7^ix^	103.13 (8)
O3^iv^—Sb2—O5^iv^	93.3 (2)	O7^xvi^—Rb2—O7^ix^	165.42 (8)
O6^vi^—Sb2—O5^iv^	78.42 (12)	O3—Rb2—O7^ix^	70.82 (10)
O6^iv^—Sb2—O5^iv^	78.42 (12)	O8^ix^—Rb2—O7^ix^	56.58 (9)
O2^vii^—Sb2—O5^iv^	164.8 (2)	O7^xvii^—Rb2—O7^ix^	84.86 (13)
O3^iv^—Sb2—O2^iv^	176.5 (2)	O7^xv^—Rb2—O4^ix^	90.66 (9)
O6^vi^—Sb2—O2^iv^	84.25 (12)	O7^xvi^—Rb2—O4^ix^	137.99 (10)
O6^iv^—Sb2—O2^iv^	84.25 (12)	O3—Rb2—O4^ix^	119.13 (9)
O2^vii^—Sb2—O2^iv^	74.6 (2)	O8^ix^—Rb2—O4^ix^	47.69 (7)
O5^iv^—Sb2—O2^iv^	90.24 (19)	O7^xvii^—Rb2—O4^ix^	103.67 (9)
O3^iv^—Sb2—Sb3^iv^	99.95 (13)	O7^ix^—Rb2—O4^ix^	48.47 (9)
O6^vi^—Sb2—Sb3^iv^	116.27 (12)	O7^xv^—Rb2—O4^xvii^	137.99 (10)
O6^iv^—Sb2—Sb3^iv^	38.97 (12)	O7^xvi^—Rb2—O4^xvii^	90.66 (9)
O2^vii^—Sb2—Sb3^iv^	135.10 (7)	O3—Rb2—O4^xvii^	119.13 (9)
O5^iv^—Sb2—Sb3^iv^	39.73 (3)	O8^ix^—Rb2—O4^xvii^	47.69 (7)
O2^iv^—Sb2—Sb3^iv^	82.79 (11)	O7^xvii^—Rb2—O4^xvii^	48.47 (9)
O3^iv^—Sb2—Sb3^vi^	99.95 (13)	O7^ix^—Rb2—O4^xvii^	103.67 (9)
O6^vi^—Sb2—Sb3^vi^	38.97 (12)	O4^ix^—Rb2—O4^xvii^	83.58 (13)
O6^iv^—Sb2—Sb3^vi^	116.27 (12)	O7^xv^—Rb2—O4^xvi^	80.00 (10)
O2^vii^—Sb2—Sb3^vi^	135.10 (7)	O7^xvi^—Rb2—O4^xvi^	49.82 (9)
O5^iv^—Sb2—Sb3^vi^	39.73 (3)	O3—Rb2—O4^xvi^	50.19 (10)
O2^iv^—Sb2—Sb3^vi^	82.79 (11)	O8^ix^—Rb2—O4^xvi^	141.55 (9)
Sb3^iv^—Sb2—Sb3^vi^	77.522 (16)	O7^xvii^—Rb2—O4^xvi^	85.45 (9)
O3^iv^—Sb2—Sb2^viii^	140.79 (17)	O7^ix^—Rb2—O4^xvi^	120.02 (9)
O6^vi^—Sb2—Sb2^viii^	92.06 (12)	O4^ix^—Rb2—O4^xvi^	163.25 (10)
O6^iv^—Sb2—Sb2^viii^	92.06 (12)	O4^xvii^—Rb2—O4^xvi^	112.65 (7)
O2^vii^—Sb2—Sb2^viii^	38.89 (14)	O7^xv^—Rb2—O4^xv^	49.82 (9)
O5^iv^—Sb2—Sb2^viii^	125.90 (15)	O7^xvi^—Rb2—O4^xv^	80.00 (10)
O2^iv^—Sb2—Sb2^viii^	35.67 (14)	O3—Rb2—O4^xv^	50.19 (10)
Sb3^iv^—Sb2—Sb2^viii^	110.286 (17)	O8^ix^—Rb2—O4^xv^	141.55 (9)
Sb3^vi^—Sb2—Sb2^viii^	110.286 (17)	O7^xvii^—Rb2—O4^xv^	120.02 (9)
O8—Sb3—O4	85.82 (18)	O7^ix^—Rb2—O4^xv^	85.45 (9)
O8—Sb3—O7^ix^	100.65 (19)	O4^ix^—Rb2—O4^xv^	112.65 (7)
O4—Sb3—O7^ix^	168.10 (15)	O4^xvii^—Rb2—O4^xv^	163.25 (10)
O8—Sb3—O7	99.2 (2)	O4^xvi^—Rb2—O4^xv^	50.88 (12)
O4—Sb3—O7	88.24 (15)	Rb2^xviii^—Rb2—Rb2^xix^	161.64 (5)
O7^ix^—Sb3—O7	80.91 (16)	Sb3^ix^—O7—Sb3	99.09 (16)
O8—Sb3—O6	92.83 (19)	Sb3^ix^—O7—Rb2^ii^	135.39 (17)
O4—Sb3—O6	95.72 (15)	Sb3—O7—Rb2^ii^	118.83 (16)
O7^ix^—Sb3—O6	93.94 (15)	Sb3^ix^—O7—Rb2^ix^	126.22 (16)
O7—Sb3—O6	167.60 (15)	Sb3—O7—Rb2^ix^	93.33 (13)
O8—Sb3—O5	167.6 (2)	Rb2^ii^—O7—Rb2^ix^	76.87 (8)
O4—Sb3—O5	88.31 (18)	Sb2^iv^—O6—Sb3	102.87 (16)
O7^ix^—Sb3—O5	87.13 (18)	Sb2^iv^—O6—Rb1^xi^	115.79 (16)
O7—Sb3—O5	91.56 (18)	Sb3—O6—Rb1^xi^	140.87 (15)
O6—Sb3—O5	76.85 (17)	Sb2^iv^—O6—Rb1	128.30 (17)
O8—Sb3—Sb3^ix^	103.08 (16)	Sb3—O6—Rb1	91.48 (12)
O4—Sb3—Sb3^ix^	128.50 (11)	Rb1^xi^—O6—Rb1	69.77 (8)
O7^ix^—Sb3—Sb3^ix^	40.47 (11)	Sb2^xx^—O2—Sb1	129.7 (2)
O7—Sb3—Sb3^ix^	40.43 (10)	Sb2^xx^—O2—Sb2^iv^	105.4 (2)
O6—Sb3—Sb3^ix^	133.40 (11)	Sb1—O2—Sb2^iv^	124.9 (2)
O5—Sb3—Sb3^ix^	89.14 (15)	Sb2^iv^—O3—Sb1^xv^	128.4 (3)
O8—Sb3—Sb2^iv^	129.85 (16)	Sb2^iv^—O3—Rb2	127.7 (2)
O4—Sb3—Sb2^iv^	89.09 (11)	Sb1^xv^—O3—Rb2	103.86 (19)
O7^ix^—Sb3—Sb2^iv^	94.24 (11)	Sb2^iv^—O5—Sb3^i^	101.30 (15)
O7—Sb3—Sb2^iv^	130.51 (11)	Sb2^iv^—O5—Sb3	101.30 (15)
O6—Sb3—Sb2^iv^	38.16 (9)	Sb3^i^—O5—Sb3	147.7 (3)
O5—Sb3—Sb2^iv^	38.97 (15)	Sb1^xxi^—O1—Sb1^iv^	99.0 (2)
Sb3^ix^—Sb3—Sb2^iv^	118.398 (18)	Sb1^xxi^—O1—Rb1^iv^	137.03 (6)
O8—Rb1—O6^x^	122.58 (10)	Sb1^iv^—O1—Rb1^iv^	108.38 (6)
O8—Rb1—O6^xi^	122.58 (10)	Sb1^xxi^—O1—Rb1^xxii^	108.38 (6)
O6^x^—Rb1—O6^xi^	76.51 (14)	Sb1^iv^—O1—Rb1^xxii^	137.03 (6)
O8—Rb1—O1^iv^	98.45 (6)	Rb1^iv^—O1—Rb1^xxii^	72.53 (11)
O6^x^—Rb1—O1^iv^	75.47 (8)	Sb3^xiii^—O8—Sb3	144.8 (3)
O6^xi^—Rb1—O1^iv^	138.39 (8)	Sb3^xiii^—O8—Rb1	100.33 (16)
O8—Rb1—O1^xii^	98.45 (6)	Sb3—O8—Rb1	100.33 (16)
O6^x^—Rb1—O1^xii^	138.39 (8)	Sb3^xiii^—O8—Rb2^ix^	99.97 (16)
O6^xi^—Rb1—O1^xii^	75.47 (8)	Sb3—O8—Rb2^ix^	99.97 (16)
O1^iv^—Rb1—O1^xii^	107.47 (11)	Rb1—O8—Rb2^ix^	108.63 (15)
O8—Rb1—O6^xiii^	54.15 (10)	Sb1—O4—Sb3	133.59 (19)
O6^x^—Rb1—O6^xiii^	110.23 (8)	Sb1—O4—Rb1	100.91 (14)
O6^xi^—Rb1—O6^xiii^	68.45 (13)	Sb3—O4—Rb1	89.24 (12)
O1^iv^—Rb1—O6^xiii^	151.31 (8)	Sb1—O4—Rb2^ix^	136.26 (15)
O1^xii^—Rb1—O6^xiii^	87.06 (8)	Sb3—O4—Rb2^ix^	87.33 (12)
O8—Rb1—O6	54.15 (10)	Rb1—O4—Rb2^ix^	92.95 (9)
O6^x^—Rb1—O6	68.45 (13)	Sb1—O4—Rb2^ii^	87.90 (12)
O6^xi^—Rb1—O6	110.23 (8)	Sb3—O4—Rb2^ii^	99.39 (14)
O1^iv^—Rb1—O6	87.06 (8)	Rb1—O4—Rb2^ii^	157.86 (12)
O1^xii^—Rb1—O6	151.31 (8)	Rb2^ix^—O4—Rb2^ii^	67.35 (7)

Symmetry codes: (i) *x*, −*y*, *z*; (ii) *x*, *y*, *z*+1; (iii) *x*+1, *y*, *z*+1; (iv) −*x*+1, −*y*, −*z*+1; (v) −*x*+2, −*y*, −*z*+2; (vi) −*x*+1, *y*, −*z*+1; (vii) *x*−1, *y*, *z*; (viii) −*x*, −*y*, −*z*+1; (ix) −*x*+3/2, −*y*−1/2, −*z*+1; (x) −*x*+2, *y*, −*z*+1; (xi) −*x*+2, −*y*−1, −*z*+1; (xii) *x*+1, *y*−1, *z*+1; (xiii) *x*, −*y*−1, *z*; (xiv) −*x*+2, −*y*−1, −*z*+2; (xv) *x*, *y*, *z*−1; (xvi) *x*, −*y*, *z*−1; (xvii) −*x*+3/2, *y*+1/2, −*z*+1; (xviii) −*x*+3/2, −*y*+1/2, −*z*; (xix) −*x*+3/2, −*y*−1/2, −*z*; (xx) *x*+1, *y*, *z*; (xxi) *x*−1, *y*, *z*−1; (xxii) *x*−1, *y*+1, *z*−1.

## References

[bb1] Brandenburg, K. (2006). *DIAMOND* Crystal Impact GbR, Bonn, Germany.

[bb2] Duhlev, R. (1994). *Acta Cryst.* C**50**, 1523–1525.

[bb3] Farrugia, L. J. (1999). *J. Appl. Cryst.* **32**, 837–838.

[bb4] Hirschle, C. J. R., Emmerling, F. & Röhr, C. (2001). *Z. Naturforsch. Teil B*, **56**, 169-178.

[bb5] Hong, H. Y.-P. (1974). *Acta Cryst.* B**30**, 945–952.

[bb6] Nonius (1998). *COLLECT* Nonius BV, Delft, The Netherlands.

[bb7] Otwinowski, Z. & Minor, W. (1997). *Methods in Enzymology*, Vol. 276, Macromolecular Crystallography, Part A, edited by C. W. Carter Jr & R. M. Sweet, pp. 307–326. New York: Academic Press.

[bb8] Sheldrick, G. M. (2007). *SADABS* University of Göttingen, Germany.

[bb9] Sheldrick, G. M. (2008). *Acta Cryst.* A**64**, 112–122. 10.1107/S0108767307043930 18156677

